# Sports nature culture. A participatory approach to reconstruct a multidimensional cultural landscape by leveraging outdoor sports

**DOI:** 10.3389/fspor.2025.1554007

**Published:** 2025-03-07

**Authors:** Barbara Camocini, Laura Daglio, Roberto Podda

**Affiliations:** ^1^ Department of Design, Politecnico di Milano, Milan, Italy; ^2^ Department of Architecture Built Environment and Construction Engineering, Politecnico di Milano, Milan, Italy; ^3^ Department of Architecture, XJTLU_Xi'an Jiaotong—Liverpool University, Suzhou, China

**Keywords:** outdoor sports, cultural landscape, rural and natural landscape enhancement, participatory design, small towns regeneration

## Abstract

The growing popularity of outdoor sports in natural landscapes is reshaping sport tourism, particularly in rural and mountainous areas, offering opportunities for sustainable development but also presenting risks to the environmental and cultural heritage. This study focuses on Oliena, a village in Sardinia (Italy) that is undergoing depopulation and economic decline, leveraging participatory approaches through Nature Sports to strengthen the community identity and the bond with its landscape. Since 2016, the team of researchers have conducted participatory design workshops and field activities, including creative installations, and multidimensional narrative of the urban and landscape heritage leveraged by Oliena's unique cultural and natural resources. Data collection involved stakeholder meetings, interviews, and sports experiences, fostering a shared understanding of the interplay between the community and its environment. This experience led to the establishment of a Permanent Workshop for continuous engagement and community capacity building. The integrated approach experienced in the case study of Oliena showcases how participatory approaches and shared sports experiences can reconnect communities with their heritage, promoting tourism that is both enriching and sustainable. It highlights how Nature Sports, integrated within cultural landscapes, can provide a transformative framework for sustainable tourism, offering a methodology with scalable insights for other regions.

## Introduction: the changing scenario of sport tourism in rural and mountainous regions

In recent years, outdoor sports activities in natural landscapes have become increasingly popular (1) driven by the emerging health and well-being culture (2) and further reinforced by a renewed need for a direct contact with nature (3), which has been prompted by the COVID-19 pandemic. Nature sports, in particular, defined as formal and informal activities, practiced in natural or rural areas (4) are in fact attracting a significant and growing number of participants.

In mountainous regions these activities have historically served as key drivers of social and economic development. In response to the market crisis that mountain tourism has long faced (5), new forms of tourism are emerging shaped by economic, societal, and environmental transformations (6, 7), also spurring from the thriving popularity of outdoor sports (8). The impacts of global warming and climate change (9) have accelerated this shift, prompting the exploration of new economic opportunities for year-round activities (10), both as an alternative to and as an integration with traditional winter sports. Conventional mountaineering, trekking, and biking have significantly evolved and diversified (11) offering new experiences, technologies, and infrastructures to meet a growing demand that is determined by cultural shifts, industry innovation and demographic changes such as those due to an ageing population (12).

Inspired by the successes of mountain resorts, other tourist destinations with access to natural resources are increasingly leveraging sports activities as a foundation for their development strategies (13). From the organization of small-scale tourism events (14) to the implementation of new infrastructures and marketing campaigns local authorities and communities in inner areas are capitalizing on these trends to foster reactivation and stimulate their economies. By taking action for nature, the sports community can significantly contribute to global efforts to achieve the United Nations Sustainable Development Goals (SDGs) and its 2030 Agenda, which recognizes sport as a potential enabler of sustainable development (15).

Nevertheless, the redevelopment of areas through sports carries dual impacts. On the one hand, it can reactivate certain regions, bringing economic prosperity; on the other hand, it can jeopardize the very natural and landscape features that originally attracted visitors. Not only it presents potential threats and environmental concerns related to increased human impact, soil erosion, and vegetation decline (16), and raises questions about the general ecological qualities of the place; but it also requires a specific attention on the complex bidirectional relationship between sport and of the natural environment (17) aimed at controlling and mitigating the potential impacts of transformation that can thus be generated.

In addition to environmental challenges, the controversial relationship between tourism and landscape encompasses also a social risk for the local population in terms of identity loss. Since the image of a place serves both as a valued resource that draws tourists and as the stage upon which tourism unfolds, a possible imbalance can be generated between the appeal of the landscape as a tourist attraction and the degradation caused by the impact of tourism (18). This tension underscores the importance of sustainable tourism practices that balance economic growth, environmental preservation, and social well-being.

Within this context, this paper explores the potential of nature sports to reactivate inland areas through community engagement aimed at promoting sustainable tourism while preventing and managing environmental and social side effects (19). Specifically, it presents the case of an ongoing research activity on Oliena (NU), a village in the northeast mountain region of Sardinia addressing the issue of depopulation and economic decline through the development of outdoor landscape-based tourism. Within the ongoing fieldwork experiments with participatory strategies to plan and manage the reactivation, various forms of participation related to nature sports are analyzed as means for potential redevelopment. A methodological toolbox is thus presented, detailing how these participatory approaches have been tested in analytical and design briefing practices that integrate landscape culture, sports, and local inhabitants. Finally, the results are discussed to reveal potential methods and approaches that may be applied in other contexts.

## The importance of participation for sustainable sport tourism

Citizen's active participation plays an essential role in pursuing environmental, economic and social sustainable changes (20). Specifically, community participation is considered a significant driver for sustainable tourism especially in areas where environmental and anthropological resources can create tourist place attachment but are affected by depopulation (21). The direct involvement of all stakeholders is essential for achieving long-lasting outcomes, since it cultivates a sense of local ownership and accountability, and a long-term environmental stewardship playing a critical role in safeguarding natural resources and encouraging responsible tourism practices (22).

Moreover, sports can serve as a powerful platform to promote pro-environmental behavior among both participants (23) and spectators highlighting the importance also of their engagement in meaningful climate action with demonstrative results (24). In particular, outdoor sports in natural settings can induce a diversity of involvement and experiences in participants (25) stemming from the relationship with the natural landscape with its immeasurable and unpredictable power (26). The special experience of awe exerts in participants an extensive influence on actual environmental responsible behavior (27).

Additionally, an enhanced cultural relationship between participants of sports and the landscape can be recognized because of their interactions with each other and with the material environment allowing for a deeper understanding of the place, strengthen their sense of belonging and develop social values sharing the experience with other people (28).

Finally, the social feature of participation in outdoor sports, that is the multidimensional interplay between participants, community and environment encompasses on the one hand cognitive experiences involving attitudes, awareness, and knowledge; on the other hand, affective responses encompassing feelings and emotions. The attunement of these can have the potential to nurture cultures of care (29).

The participatory approaches developed for the exploration of the reactivation of the town of Oliena were adopted in various phases of the process, from initial analysis to the definition of the brief, as well as the design and management stages. The organization of nature sport activities in the landscape of Oliena territory in the workshop editions over the years, as well as meetings and interviews with the local sports communities have played a crucial role in understanding the deep relationship between community and place (30) and fathom the landscape as a historical layering of collective values. In addition, the shared activities in nature among experts, researchers, and inhabitants, has allowed for raising awareness, and sharing the affective relationship with the place, restructuring connections aimed at recognizing the complex system of relationships between community nature and built environment.

## The Oliena context: the decline of a tangible and intangible rural culture

The village of Oliena, in the province of Nuoro, is located at the foot of Mount Corrasi, part of the Supramonte mountain chain in Barbagia, a central area of the island of Sardinia, Italy. It is immersed in a landscape of great natural, geological, historical, morphological, and archaeological value, shaped over the long term by an ancient rural culture.

The Lanaitto valley and the Cedrino river, into which the waters of the Su Gologone springs flow, are shaped by the Supramonte, a limestone chain carved out by karst phenomena that have created tunnels and numerous caves. Closely linked to the nature of this landscape and its water resources are significant pre-Nuragic settlements.

The fertile valleys, shaped since ancient times into a historic rural landscape, support a thriving agricultural production of wine and oil. Consequently, tourism in the region can be strengthened by both the strong identity value of the territory and the uniqueness of its natural landscape. This combination facilitates the practice of a wide and growing variety of nature sports, alongside cultural visits, culinary and handicraft experiences, and the warm hospitality of the local population.

However, Oliena is undergoing a process of decline and depopulation leading to economic stagnation and the abandonment of the historic center. The strong ancient rural culture although it has changed and adapted over time, has become outdated, leading to a process of gradual loss of the identity and meanings that once derived from the symbiosis between rural life and the landscape to which it was connected, of the link between the built environment and its surrounding territory. This cultural fragmentation requires a systemic re-composition that can benefit from the new values and scenarios brought about by contemporary society, triggered by the rich diversity of the natural landscape and the possible nature sports offer.

## Setting the methodological approach

The activities here presented were developed since 2016 by a team of researchers from Politecnico di Milano in collaboration with International Universities and local associations, aimed at exploring the potential and feasibility of reactivating the village. The involvement of the inhabitants alternated with preparatory phases of remote university research, which supported the organization of on-site activities (round tables, workshops, exhibitions) lasting 10 to 15 days each year (excluding pandemic years), culminating in a final presentation event at the local Cortes Apertas festival, which attracts thousands of people every September. The objective was to explore possible models of urban and territorial regeneration and valorization, relying on a continuous participatory approach that engages local citizens.

The importance of outdoor sports, particularly those related to the distinctive features of the local landscape (sky-racing, free climbing, caving, hiking, etc.), emerged during the surveys of the Oliena area, which were planned as part of the on-site workshops. These sports activities served as spontaneous aggregating factors for the local community, fostering a shared connection and appreciation for their landscape. This engagement stimulated the researchers and supplemented the traditional historical narrative of the area, with outdoor sports often becoming central to communication between the team of researchers and students and the local population.

**Figure 1:**
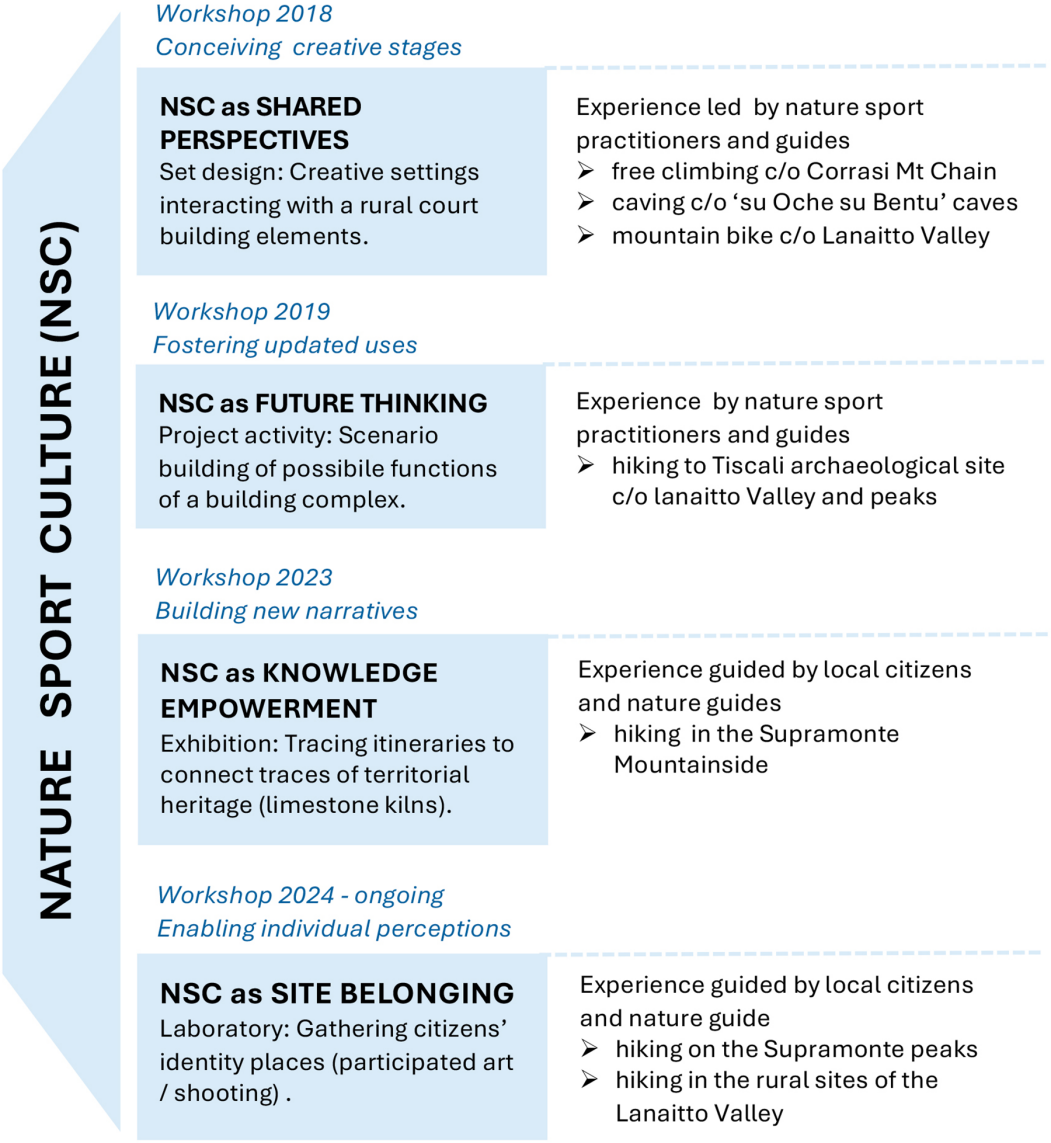
Set of nature sports strategies experimented within the research.

Various narrative strategies were experimented with in the participatory activities conducted as part of the ongoing research (cf. Figure 1).

### Sport as common perspective: creative staging

A Participatory Design workshop was organized aimed at the setting up of creative installations in a small abandoned ancient courtyard building in the historic center of Oliena, designed with the involvement of the population focusing on the relationship with the past and present natural landscape.

**Figure 2:**
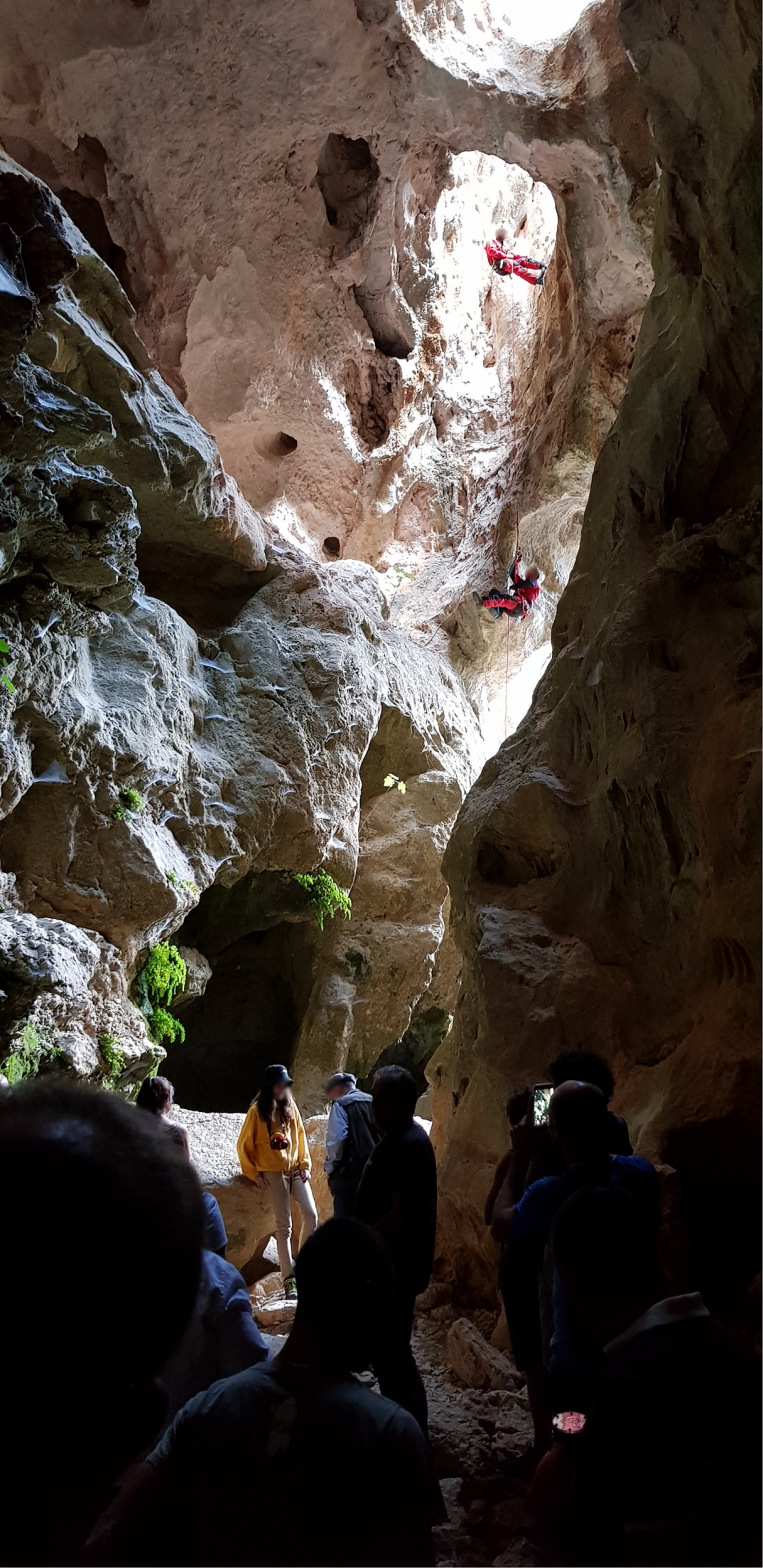
Shared sports experience in the “Su Oche Su Bentu” caves.

The faculty team coordinated the ten-day workshop activities, during which students and young researchers, along with citizens, participated in meetings with stakeholders, and evening seminars with guest experts who explored various aspects of local culture. In particular, the participants took part in hiking activities in the surrounding natural territory, accompanied by professional guides and nature sports practitioners, such as cave climbers in visits to the “Su Oche Su Bentu” caves (cf. Figure 2), free climbers at the foot of the cliffs of Mount Corrasi, and bikers and runners in walks along the Supramonte peaks.

This immersive experience, involving close interaction with Nature Sports practitioners, allowed for a continuous exchange of knowledge and interpretive perspectives on both present and past realities, stimulating the adoption of novel modes of observation and storytelling, fostering the sharing of experiences and approaches within a common ground between guests and local participants. The resulting creative installations offered a new understanding of the nature-culture nexus through sports practiced collaboratively by foreign designers and local participants. This process fostered the development of a renewed relationship between the village and its natural landscape.

### Sport as future thinking: scenario building projects

The study of local heritage informed the regeneration projects of an urban block in the historic center of Oliena aimed at reconnecting with the territory through the design of new uses, such as places for training, research centers, performance, and sports facilities.

The design process was characterized by a phase of brief construction and scenario building (31, 32) resulting from an immersive experience led through explorative trails in the urban and rural natural territory, at archaeological sites in the area, and interviews with stakeholders and experts. With the aim of promoting places that hold evidence of local historical heritage, scattered throughout the territory and often within walking distance, the need to define and systematize the itineraries and strengthen the local structures for formation and training tourist guides that allow their fruition raised as an emergency. Focusing on the relationship between the territory and the historic built environment, which has shaped the rich tangible and intangible cultural heritage of the area over time, these activities emphasized the importance of integrating nature sports with the region's extensive cultural offerings to promote a more comprehensive approach to slow tourism development in Oliena.

### Sport as knowledge empowerment: itineraries merging multifaceted layers of landscape

Following the analytical, interpretive, and scenario building phases, the research explored the design of a new system of trails within the landscape, highlighting the distinctive limestone geological features that define the area's identity. The development of the new itineraries, bridging nature with historical memory, and the empirical culture of the area, was made possible thanks to the involvement of the local population.

The team from Politecnico di Milano, in collaboration with young graduates, collected and stimulated both tangible and intangible testimonies of traditional lime production and conducted guided excursions along the slopes of Supramonte mountain range to locate and survey the remains of lime kilns and simultaneously gaining insights into the geological composition of the area. These initial accounts of Oliena's socio-economic history were organized, co-designed, and conveyed in narrative form through an exhibition (entitled “Dae Predaes Nostras”) set-up in the courtyard of the former Jesuit College, a site of significant historical importance for the village. The exhibition provided the public with a preliminary visualization model of a potential hiking route system that connects the area's natural and cultural heritage.

### Sport as perception site belonging: paths of awareness

The final ongoing participated activity was conceived with two primary objectives: to strengthen community awareness and foster a sense of belonging thereby cultivating a culture of care; and to identify and map areas and places that evoke memories, emotions, and attachments among the inhabitants.

In fact, a photographic survey was launched, inviting citizens to organize hikes through the landscape to capture their favorite spots within the territory—places that resonate with their personal or family history and identity. The workshop, entitled “Inohe so. Here I recognize myself between home and land” aims to explore the affective landscape of Oliena through the eyes of its inhabitants who take photographic shots accompanied by a short personal story explaining the significance of their chosen locations. A final exhibition is planned for September to share and expand this participatory process across the broader mountain region of Barbagia. This activity seeks to both enhance and diversify the hiking trail itineraries and identify unique destinations that deepen the connection between tourists and residents. The resulting augmented cultural and social experience will strengthen the relationship between visitors and the community while promoting a deeper appreciation of Oliena's cultural and natural heritage.

## Preliminary results and discussion

Over the years, the participatory tools and activities employed in this research have explored diverse approaches to fostering relationships between the community, external participants, and the environment through shared nature sports practices as a means of exchanging information, feelings, and emotions. Adopted in analytical and interpretive stages, including scenario building and co-design explorations, these activities and related events have ultimately raised awareness of the landscape as a pillar of community identity (33) and its full potential for the development of future nature sports, while simultaneously engaging the community in local regeneration efforts (34).

Although the research and design activities were conducted over multiple years, they have generated increasing interest and participation from the local community, particularly through various associations, eventually prompting the Municipality's commitment to enhancing the area through networking and fundraising efforts. In fact, in the final year, a Permanent Workshop was established to support participatory activities and institutionalize the gradual transfer of autonomy to the engaged population, envisioning the social impact of new sports events on the area's regeneration (35). The Permanent Workshop serves two primary objectives: first, to reinstate local community involvement in organizing planned activities and events as a means of fostering socially sustainable behaviors (36); second, to empower the community through training initiatives. A particular focus is placed on enabling younger generations to benefit from the potential of sustainable tourism development in the area (37) and encouraging them to remain in their hometown.

However, only recently was the Permanent Workshop created following a direct action by the Oliena Municipality. Transformative effective outcomes require a broader dialogue among diverse stakeholders from both private and public sectors, operating across multiple institutional levels (38). The integration of these actors facilitates a multiscalar, integrated perspective, merging top-down and bottom-up initiatives, crafting a shared narrative, and co-creating a future vision (39). Moreover, reinforcing the recognized interconnections between the Olianese landscape and the broader Barbagia region can foster political involvement to support through new focused policies and fund the regeneration, merging local networks and community groups actions with top-down measures and initiatives (40), possibly even on an international scale (41).

Finally, the activities organized over these years have provided two key insights. First, the successful development of a tourist destination depends on understanding the multilevel complexity of the initiative (42), on moving beyond a sectoral focus, such as merely developing new infrastructures and facilities. Secondly, success hinges on fully articulating the natural and cultural assets of the area by leveraging its unique endogenous resources creating synergies between nature sports and slow tourism experiences, recognizing the complex set of resulting emotions to enhance the attractiveness of the place (43), fostering a dynamic interplay between the “experience” of physical activity and the enjoyment of local products and complementary services (44).

## Conclusions

The research aimed at establishing a strong connection between the natural, cultural, and sports dimensions of the landscape. This has been made possible through the contribution of the community, which helped to uncover and understand the historical, multidimensional character of the region and facilitated a contemporary reinterpretation of the area, enriched by its diverse tangible and intangible features. The experiences and the participatory tools applied have yielded results that can be scaled and adapted to other case studies, offering valuable methodological insights, despite some limits.

In fact, although preceded by intense preliminary research, the fieldwork of the research group was conducted for ten days each year in proximity to the Barbagia autumn festival, which limited the potential for continuous community engagement, until the recent establishment of the Permanent Workshop, resulting also from the direct involvement of the Municipality.

Moreover, since the process is still ongoing, positive outcomes from the participatory tools tested can be recognized but mainly stemming from a planning to design and management phase and the results in terms of regeneration driven by the wider adoption of nature sports tourism and its sustainable management have yet to be assessed.

However, based on the accumulated experience, the adopted methodologies allow for the formulation of some programmatic recommendations linked by a common systemic approach. First, the study highlights the importance of establishing cross-category relationships among policymakers and stakeholders ideally from the early stages of the process to enhance its effectiveness by strengthening the integration of bottom-up and top-down initiatives.

Secondly, considering the multidimensional and multidisciplinary nature of the regeneration process, encouraging synergies and cross-category relationships among different landscape features, as explored through research, has demonstrated significant potential in supporting transformation while simultaneously addressing its diverse aspects.

This systemic approach can lead to a rich and diversified offering, which, from an operational perspective, can be expressed through the concept of a “tourism portfolio.” Commonly associated with sports events (45, 46), this approach enhances the tourist experience, strengthens place marketing, and ensures sustainability by utilizing resources in a synergistic manner.

Considering the aforementioned needs, future research will focus on mapping the landscape from the perspective of local inhabitants, incorporating the cultural and natural dimensions that, through the nature-culture approach described in this study, are linked to sports in an increasingly layered nexus. In this sense, the meaning of sports tourism (47) is enriched to emphasize not only the natural and cultural uniqueness of the place but also its social distinctiveness, enabling visitors to embark on “a psychological and perceptual journey, rather than a journey at a geographical scale” (48).

## Data Availability

The original contributions presented in the study are included in the article, further inquiries can be directed to the corresponding author.

## References

[1] Weed M Bull C. Sports Tourism: Participants, Policy and Providers. 1st ed. London: Routledge (2009).

[2] Niccolini F Barborak J Azara I Michopoulou E Cavicchi A. eds. Nature-Based Tourism and Wellbeing: Impacts and Future Outlook. Wallingford: CABI (2024).

[3] Barton J Bragg R Wood C Pretty J. eds. Green Exercise: Linking Nature, Health and Well-being. 1st ed. London and New York: Routledge (2016).

[4] Melo R Van Rheenen D James Gammon S. Part I: nature sports: a unifying concept. Ann Leis Res. (2020) 23(1):1–18. 10.1080/11745398.2019.1672307

[5] Bourdeau P. Les Sports D’hivers en Mutation: Crise ou Révolution Géoculturelle? Paris: Hermes Science Publications (2007).

[6] Pröbstl U Richins H Türk S. Winter Tourism: Trends and Challenges. Wallingford: CABI (2019).

[7] Tuppen J Langenbach M. Les territoires touristiques et sportifs en transition. Géocarrefour. (2021) 95(2). 10.4000/geocarrefour.17750

[8] Higham J Vada S. The state-of-the-art in sport tourism geographies. Tour Geogr. (2024):1–14. 10.1080/14616688.2024.2373875

[9] Steiger R Knowles N Pöll K Rutty M. Impacts of climate change on mountain tourism: a review. J Sustain Tour. (2022) 32(9):1984–2017. 10.1080/09669582.2022.2112204

[10] Bausch T Gartner WC. Winter tourism in the European Alps: is a new paradigm needed? J Outdoor Recreat Tour. (2020) 31:100297. 10.1016/j.jort.2020.100297

[11] Durán-Sánchez A Álvarez-García J del Río-Rama MC. Nature sports: state of the art of research. Ann Leis Res. (2019) 23(1):52–78. 10.1080/11745398.2019.1584535

[12] Mueller KE Thomas A. Successful aging and older adults’ health outcomes through outdoor-based interventions like adventure therapy: a scoping review. J Outdoor Environ Educ. (2024). 10.1007/s42322-024-00177-1

[13] Higham J Hinch T. Sport Tourism Development. Bristol: Channel View Publications (2018).

[14] Van Rheenen D Sobry C Melo R. Running tourism and the global rise of small-scale sport tourism events. In: Melo R Sobry C Van Rheenen D, editors. Small Scale Sport Tourism Events and Local Sustainable Development. Sports Economics, Management and Policy. Cham: Springer (2021). p. 18. 10.1007/978-3-030-62919-9_1

[15] Sherry E Agius C Topple C Clark S. Measuring Alignment and Intentionality of Sport Policy on the Sustainable Development Goals. Melbourne: Commonwealth Secretariat and Swinburne University of Technology (2020). 10.25916/sut.26252243.v1

[16] Marković JJ Petrović MD. Sport and recreation influence upon mountain area and sustainable tourism development. J Environ Tour Anal. (2013) 1(1):81–90. https://doaj.org/article/15bbe4702b1a4c42b22e457793b179a1

[17] McCullough BP Orr M Kellison T. Sport ecology: conceptualizing an emerging subdiscipline within sport management. JSport Manag. (2020) 34(6):509–20. 10.1123/jsm.2019-0294

[18] Corbisiero F De Joanna P. Tourism and landscape: conflicts, cooperation and resilience. In: Stanganelli M Gerundo C, editors. Landscape at Risk. SMC Magazine (Special Issue 4). Napoli: Luciano Editore (2020). p. 82–4.

[19] Eriksson A. ‘If they touch our cloudberries, that means war’: rural liveability and acceptance of environmental impacts from event tourism. Tour Stud. (2023) 23(4):335–51. 10.1177/14687976231200902

[20] Manzini E Rizzo F. Small projects/large changes: participatory design as an open participated process. CoDesign. (2011) 7(3–4):199–215. 10.1080/15710882.2011.630472

[21] Basile G Tani M Sciarelli M Ferri MA. Community participation as a driver of sustainable tourism: the case of an Italian village, Marettimo Island. Sinergie Italian J Manag. (2021) 39(1):81–102. 10.7433/s114.2021.06

[22] Tiwari S Marahatta D Devkota H. Aspects of community participation in eco-tourism: a systematic review. J Multidiscip Res Adv. (2024) 2(1):71–9. 10.3126/jomra.v2i1.66650

[23] Powers SL Trauntvein N. Local nature-based recreation as a pathway to environmental citizenship. J Outdoor Recreat Tour. (2024) 47:100810. 10.1016/j.jort.2024.100810

[24] McCullough BP. Advancing sport ecology research on sport and the natural environment. Sport Manag Rev. (2023) 26(5):813–33. 10.1080/14413523.2023.2260078

[25] Breivik G. Trends in adventure sports in a post-modern society. Sport Soc. (2010) 13(2):260–73. 10.1080/17430430903522970

[26] Margaryan L Fredman P. Fantastic, magical and grandiose: nature’s role in event design. In: Fredman P and Haukeland J, editors. Nordic Perspectives on Nature-based Tourism. From Place-based resources to Value-added experiences. Cheltenham: Edward Elgar (2020). p. 237–49.

[27] Wang L Jiaying L. Inspiring awe through tourism and its consequence. Ann Tour Res. (2019) 77:106–16. 10.1016/j.annals.2019.05.005

[28] Bortolotti A. Perspectives on outdoor sports: uncertainty between nature and culture. J Phys Educ Sport. (2021) 21(Supplement issue 1):638–42. 10.7752/jpes.2021.s1075

[29] Trendafilova S Ziakas V. Sensitizing the social-ecosystems of outdoor sport environments: a comprehensive framework. Front Sports Act Living. (2022) 4:937765. 10.3389/fspor.2022.937765 36051967 PMC9424613

[30] Relph E. Place and Placelessness. London: Pion (1976).

[31] Carroll JM. The scenario perspective on system development. In: Carroll JM, editor. Scenario-Based Design: Envisioning Work and Technology in System Development. New York: John Wiley (1995). p. 1–18.

[32] do Prado Leite J Hadad G Doorn J Kapla GN. A scenario construction process. Requirements Eng. (2000) 5:38–61. 10.1007/PL00010342

[33] Son IS Krolikowski C. Developing a sense of place through attendance and involvement in local events: the social sustainability perspective. Tour Recreat Res. (2024):1–12. 10.1080/02508281.2024.2335749

[34] de Luca C López-Murcia J Conticelli E Santangelo A Perello M Tondelli S. Participatory process for regenerating rural areas through heritage-led plans: the RURITAGE community-based methodology. Sustainability. (2021) 13:5212. 10.3390/su13095212

[35] Đurkin Badurina J Perić M Vitezić V. Potential for the regeneration of rural areas through local involvement in the organisation of sport events. Manag Sport Leis. (2020) 26(5):377–94. 10.1080/23750472.2020.1829990

[36] Stevenson N. The contribution of community events to social sustainability in local neighbourhoods. J Sustain Tour. (2020) 29(11–12):1776–91. 10.1080/09669582.2020.1808664

[37] Mair J Smith A. Events and sustainability: why making events more sustainable is not enough. J Sustain Tour. (2021) 29(11–12):1739–55. 10.1080/09669582.2021.1942480

[38] Romeo R Russo L Parisi F Notarianni M Manuelli S Carvao S. UNWTO. Mountain Tourism—towards a More Sustainable Path. Rome: FAO (2021). 10.4060/cb7884en

[39] Heslinga J Groote P Vanclay F. Towards resilient regions: policy recommendations for stimulating synergy between tourism and landscape. Land. (2020) 9(2):44. 10.3390/land9020044

[40] Ahlmeyer F Volgmann K. What can we expect for the development of rural areas in Europe? Trends of the last decade and their opportunities for rural regeneration. Sustainability. (2023) 15:5485. 10.3390/su15065485

[41] Georgios C Nikolaos N Michalis P. Neo-endogenous rural development: a path toward reviving rural Europe. Rural Sociol. (2021) 86:911–37. 10.1111/ruso.12380

[42] Bazzanella F Schnitzer M Peters M Bichler BF. The role of sports events in developing tourism destinations: a systematized review and future research agenda. J Sport Tour. (2023) 27(2):77–109. 10.1080/14775085.2023.2186925

[43] Hofmann V Happ E Scholl-Grissemann U. Sport destination development—insights from alpine hikers. Ger J Exerc Sport Res. (2025) 55:14–24. 10.1007/s12662-024-01011-y

[44] Radicchi E. Tourism and sport: strategic synergies to enhance the sustainable development of a local context. Phys Cult Sport Stud Res. (2013) 57(1):44–57. 10.2478/pcssr-2013-0007

[45] Ziakas V. Leveraging sport events for tourism development: the event portfolio perspective. J Glob Sport Manag. (2020) 8(1):43–72. 10.1080/24704067.2020.1731700

[46] Getz D. Event tourism: definition, evolution, and research. Tour Manag. (2008) 29(3):403–28. 10.1016/j.tourman.2007.07.017

[47] Schlemmer P Barth M Schnitzer M. Research note sport tourism versus event tourism: considerations on necessary distinction and integration. J Conv Event Tour. (2020) 2:91–9. 10.1080/15470148.2019.1710314

[48] Weed M. The geographies and psychologies of global transitions: implications for sport, tourism, climate, health, wellbeing, and the economy. Géocarrefour. (2021) 95(2). 10.4000/geocarrefour.19439

